# Chitosan-Coated Polymeric Silver and Gold Nanoparticles: Biosynthesis, Characterization and Potential Antibacterial Applications: A Review

**DOI:** 10.3390/polym14235302

**Published:** 2022-12-04

**Authors:** Md. Amdadul Huq, Md. Ashrafudoulla, Md. Anowar Khasru Parvez, Sri Renukadevi Balusamy, Md. Mizanur Rahman, Ji Hyung Kim, Shahina Akter

**Affiliations:** 1 Department of Food and Nutrition, College of Biotechnology and Natural Resource, Chung-Ang University, Anseong-si 17546, Gyeonggi-do, Republic of Korea; 2 Department of Food Science and Technology, Chung-Ang University, Anseong-si 17546, Gyeonggi-do, Republic of Korea; 3 Department of Microbiology, Jahangirnagar University, Savar, Dhaka 1342, Bangladesh; 4 Department of Food Science and Technology, Sejong University, Seoul 143-747, Republic of Korea; 5 Department of Biotechnology and Genetic Engineering, Faculty of Biological Science, Islamic University, Kushtia 7003, Bangladesh; 6 Department of Food Science and Biotechnology, Gachon University, Seongnam 461-701, Republic of Korea

**Keywords:** chitosan-coated silver and gold nanoparticles, biosynthesis, antibacterial applications, multidrug-resistant pathogenic bacteria

## Abstract

Biosynthesized metal nanoparticles, especially silver and gold nanoparticles, and their conjugates with biopolymers have immense potential in various fields of science due to their enormous applications, including biomedical applications. Polymeric nanoparticles are particles of small sizes from 1 nm to 1000 nm. Among different polymeric nanoparticles, chitosan-coated silver and gold nanoparticles have gained significant interest from researchers due to their various biomedical applications, such as anti-cancer, antibacterial, antiviral, antifungal, anti-inflammatory technologies, as well as targeted drug delivery, etc. Multidrug-resistant pathogenic bacteria have become a serious threat to public health day by day. Novel, effective, and safe antibacterial agents are required to control these multidrug-resistant pathogenic microorganisms. Chitosan-coated silver and gold nanoparticles could be effective and safe agents for controlling these pathogens. It is proven that both chitosan and silver or gold nanoparticles have strong antibacterial activity. By the conjugation of biopolymer chitosan with silver or gold nanoparticles, the stability and antibacterial efficacy against multidrug-resistant pathogenic bacteria will be increased significantly, as well as their toxicity in humans being decreased. In recent years, chitosan-coated silver and gold nanoparticles have been increasingly investigated due to their potential applications in nanomedicine. This review discusses the biologically facile, rapid, and ecofriendly synthesis of chitosan-coated silver and gold nanoparticles; their characterization; and potential antibacterial applications against multidrug-resistant pathogenic bacteria.

## 1. Introduction

In recent years, bio-nanotechnology has attracted remarkable attention from researchers due to its extensive usage in different fields of science, especially for developing new bioactive materials. Metal nanoparticles are small particles with a large surface area, making them perfect for utilization in various biomedical and industrial sectors. Among different types of metal nanoparticles, silver and gold nanoparticles have gained significant attention due to their wide range of applications in various fields of science. In recent years, they have been widely used to develop antibacterial, antifungal, antiviral, and anticancer technologies, as well as gene therapy agents, biosensors, drug delivery, chronic disease diagnostics systems, etc. [1,2,3,4,5,6,7]. Recent studies have shown the vigorous antimicrobial activity of silver nanoparticles (AgNPs) against numerous pathogenic microorganisms, including multidrug-resistant bacteria [1,8,9]. AgNPs are often added to topical creams, hand gel, medical catheter coverings, wound dressings, antiseptic sprays, and cosmetics, etc., due to their effective antimicrobial properties [10,11,12]. Several reports have described the applications of AgNPs as wound-healing agents [13,14]. AgNPs were also effectively used as vehicles for various drugs to treat different diseases [15,16,17,18]. Gold nanoparticles (AuNPs) are also extensively used in biomedical science due to their high functionality, ease of detection, biocompatibility, and low toxicity [1,19,20]. Many studies have investigated numerous applications of AuNPs as drug and gene delivery agents to treat different diseases and as antimicrobial agents to control pathogenic microorganisms [1,21,22,23,24,25].

Polymer-coated metallic nanoparticles have gained considerable interest over recent years due to their unique physicochemical properties and wide applications. Among various polymeric nanoparticles, chitosan-coated polymeric silver and gold nanoparticles represent an emerging group of bioactive hybrid materials in medical science because of their biodegradability, biocompatibility, high activity, and stability with low toxicity [26,27]. Chitosan (Ch) is a biopolymer and is considered a non-toxic polymer that shows excellent antibacterial and antifungal activities against numerous pathogenic microorganisms, compared to other bioactive polymers [27,28]. There are many reports on the application of Ch inhibiting the growth of pathogenic microorganisms, including both Gram-positive and Gram-negative bacteria [29,30,31]. Ch is a natural polysaccharide and is widely used in pharmaceutical industries, as well as food industries due to its high biocompatibility and biodegradability with low toxicity [32,33]. Commercially, the bioactive polymer Ch is synthesized through the deacetylation process of chitin, which is collected from the outer skeleton of crab, shrimp, lobster, and crayfish shells [34]. Structurally, Ch is a cationic biopolymer consisting of D-glucosamine and N-acetyl D-glucosamine units attached by β-1,4 glycosidic bonds. Biopolymer Ch has two types of bioactive functional groups, the hydroxyl group and the amino group, and these active groups are responsible for the potential antimicrobial activity of Ch [34,35]. Ch is a positively charged molecule due to the presence of –NH^3+^ groups, and these active amino groups are also responsible for the interaction with the negatively charged cell membranes of bacteria [27,35]. Ch is also used as a stabilizing agent for the synthesis of different metallic nanoparticles. It can facilitate the modification of the surface physical absorption and electrostatic interaction, thus improving the stability and bioactivity of nanoparticles and making them a perfect candidate as potential therapeutic agents [36,37,38,39].

The emergence of multidrug-resistant (MDR) pathogenic bacteria seriously threatens public health worldwide [40]. The MDR bacteria create different health problems, including infectious diseases and threats to decrease the yield of many accomplishments, such as surgical procedures, transplantation, cancer care, etc. [41]. These MDR bacteria include *Staphylococcus aureus*, *Klebsiella pneumoniae*, *Streptococcus pneumoniae*, *Escherichia coli*, *Acinetobacter baumannii*, *Pseudomonas aeruginosa*, *Vibrio parahaemolyticus*, *Salmonella* Typhimurium, *Enterococcus faecium*, *Enterococcus faecalis*, *Enterobacter* spp., etc. [36,42,43]. Day by day many other bacteria also increasingly becoming resistant to antibiotics. Therefore, the development of new, safe, and effective antibacterial agents is urgently required. Many recent studies showed the efficacy of silver and gold nanoparticles in controlling multidrug-resistant microorganisms [1,2,44,45]. However, the main drawbacks of these nanoparticles are low stability and high toxicity [46,47]. By the conjugation of Ch with silver nanoparticles or gold nanoparticles, the toxicity will be decreased, but the stability and efficacy will be increased significantly [46,47]. Already, several reports showed the high stability and improved efficacy of chitosan-coated silver nanoparticles (Ch-AgNPs) and chitosan-coated gold nanoparticles (Ch-AuNPs) against pathogenic bacteria [46,47]. In this review, we discuss the facile, non-toxic, and eco-friendly method for the synthesis of Ch-AgNPs and Ch-AuNPs, their characterization, and potential antibacterial applications in controlling multidrug-resistant pathogenic bacteria.

## 2. Biosynthesis of Ch-Coated Polymeric Silver and Gold Nanoparticles

There are several chemical and biological methods that are commonly applied for the synthesis of silver and gold nanoparticles and their nanocomposites. Most chemical methods are conducted using different toxic chemicals and produce various hazardous by-products [48,49]. On the other hand, biological methods use eco-friendly and nonhazardous biological agents without using any toxic chemicals [1,12]. Due to the numerous drawbacks of chemical methods, scientists are focusing more on biological techniques for facile, non-toxic, and eco-friendly synthesis of nanoparticles and nanocomposites. Moreover, biosynthesized nanoparticles and nanocomposites are pharmacologically more active than chemically synthesized nanomaterials [50]. According to Ghetas et al. [50], biologically synthesized nanoparticles showed significantly high antibacterial and antifungal activities against various pathogenic bacteria and fungi, compared to the chemically synthesized nanoparticles. The biosynthesis of nanoparticles is an eco-friendly process that uses natural compounds from plants or microbes as reducing and stabilizing agents instead of hazardous chemicals. Various biological resources, including different microbes, such as bacteria, fungi, algae, etc., and different parts of a plant, such as roots, leaves, and fruit, etc., could be utilized for the biosynthesis of nanoparticles, as well as nanocomposites [1,45,51,52].

**Figure 1:**
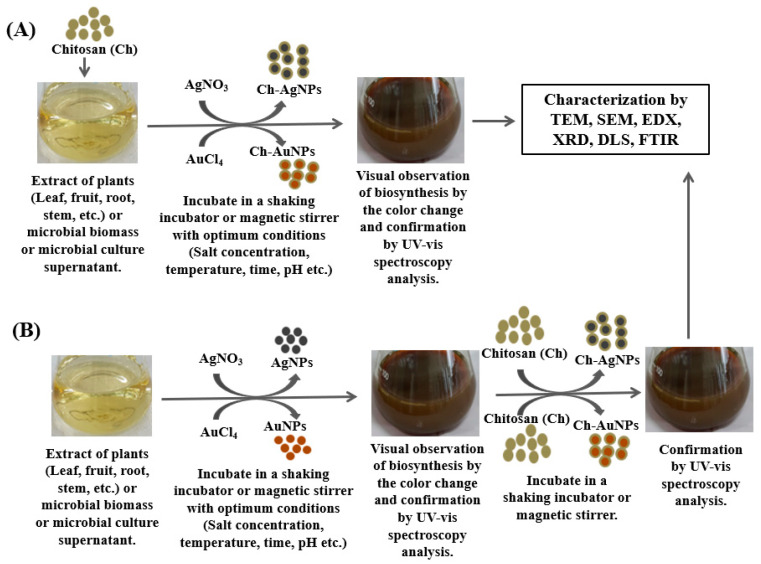
Schematic illustration of the biological synthesis of chitosan-coated polymeric silver and gold nanoparticles using plants and microbes. (**A**) One-step process, (**B**) two-step process.

There are two common biological approaches for the synthesis of Ch-AgNPs and Ch-AuNPs, a one-step process and a two-step process. In the one-step process, all materials, including plant or microbial extracts, Ch, and metal salts, such as silver nitrate (AgNO_3_) or gold (III) chloride trihydrate (HAuCl_4_·3H_2_O), are added together in a reaction flask and kept in a magnetic stirrer or in a shaking incubator with optimum conditions until the production of nanoconjugates (Figure 1A). In the two-step process, firstly, silver or gold nanoparticles are synthesized using metal salts and plant extracts or microbial culture supernatant. Then, the Ch is added to the synthesized silver or gold nanoparticles and kept in a magnetic stirrer until the production of Ch-coated polymeric silver or gold nanoparticles (Figure 1B). Paulkumar et al. [51] reported the one-step protocol for the biosynthesis of Ch-AgNPs using the stem extract of *Saccharum officinarum*. Saha et al. [47] also reported the one-step protocol for the biosynthesis of Ch-AuNPs using black pepper (*Piper nigrum*) extract. Rajeshkumar et al. [53] mentioned the two-step protocol for the green synthesis of Ch silver nanocomposites using the leaf extract of *Cissus arnottiana*. Raza et al. [54] also reported the two-step protocol for the biosynthesis of Ch-AgNPs using the cell-free extract of a fungal isolate, *Aspergillus fumigatus* KIBGE-IB33. Figure 1 illustrates the different steps of the biosynthesis of Ch-coated polymeric silver and gold nanoparticles using plants and microbes.

### 2.1. Plant-Mediated Biosynthesis

For the biosynthesis of Ch-coated polymeric silver and gold nanoparticles, different plants and their various parts, including leaf, root, shoot, stream, fruit, etc., can be used efficiently. Plant extracts are renewable and nontoxic and are prepared using an environmentally friendly aqueous medium; they require mild reaction conditions without producing any toxic byproducts [55]. Plant extracts contain various bioactive phytochemicals, such as terpenoids, flavonoids, alkaloids, polysaccharides, phenols, organic acids, vitamins, and minerals, as well as various enzymes, amino acids, and proteins [52,56,57]. These bioactive phytochemicals can be used as both reducing and stabilizing agents, as well as capping agents during the synthesis of nanoparticles and nanocomposites [52,56,57]. There are several recent reports about the facile, non-toxic, eco-friendly biosynthesis of Ch-coated polymeric silver and gold nanoparticles using different plants. For example, the leaf extract of *Cissus arnottiana* was used for the biosynthesis of Ch-AgNPs [53]. This is a two-step process, where the author first synthesized AgNPs using leaf extract of *Cissus arnottiana* as reducing and stabilizing agents. Then, the biosynthesized AgNPs were added to the Ch solution in a reaction flask and kept in a magnetic stirrer. Then, the synthesized nanocomposite pellet was collected by centrifugation and lyophilized it. Finally, the lyophilized Ch-AgNPs were dissolved in distilled water and used for characterization [53]. In another study, the plant extract of *Cuscuta reflexa* was used for the green synthesis of AgNPs, and then the Ch and synthesized AgNPs were mixed and kept in a stirrer under dark conditions. Finally, the solution was lyophilized and utilized for characterization and biomedical applications [58]. Shinde et al. [46] synthesized Ch-AgNPs using a one-step process. They added the leaf extract of *Prunus cerasus* and Ch solution in a reaction vessel, and then silver nitrate solution was added dropwise to the reaction vessel while it was magnetically stirred. Within 2 h of incubation, the reaction mixture changed from colorless to dark yellowish brown. The color change indicates the formation of Ch silver nanocomposites. Then, the reaction mixture was lyophilized to obtain the powder of Ch-AgNPs. Paulkumar et al. [51] also used the one-step process for the biosynthesis of Ch-AgNPs. A amount of 1 mM of silver nitrate was mixed with the Ch solution using a magnetic stirrer. Then, the stem extract of *S. officinarum* was added to the Ch silver nitrate suspension. After adding the stem extract, the colorless reaction mixture turned brown which indicates the synthesis of nanocomposites. The SEM and EDS analysis confirmed the formation of Ch-AgNPs [51]. Saha et al. [47] reported the biosynthesis of Ch-AuNPs using black pepper (*Piper nigrum*) extract. The biosynthesis of Ch-coated polymeric silver and gold nanoparticles and their antimicrobial applications are shown in Table 1.

### 2.2. Microbe-Mediated Biosynthesis

For the biosynthesis of Ch-coated polymeric silver and gold nanoparticles, different microbes, such as bacteria, yeast, fungi, algae, etc., can also be used. These microorganisms are wonderful biological agents for the non-toxic, cost-effective, eco-friendly, and facile synthesis of nanoparticles and nanocomposites [2,45,56]. Microbial cells or cell-free culture supernatants contain many bioactive compounds, including amino acids, proteins, enzymes, flavonoids, organic materials, and many other primary and secondary metabolites [8,56]. These biomolecules of microorganisms serve as the reducing agents and the capping and stabilizing agents during synthesizing nanoparticles and nanocomposites [8,56]. There are some recent reports about the facile, non-toxic, eco-friendly biosynthesis of Ch-coated polymeric silver and gold nanoparticles using microorganisms. For example, the cell-free extract of fungi was utilized for the biosynthesis of silver-based Ch nanocomposites [54]. Initially, they used the cell-free filtrate of the fungal isolate for the biosynthesis of AgNPs. Then, the pre-synthesized AgNPs and Ch solution were mixed and the bioactive Ch-AgNPs were formed under microwave irradiation. The presence of hydroxyl and amino groups on the biopolymer Ch influence the formation of nanocomposites by binding the metallic components of the metal ions [54]. Youssef et al. [71], also used a two-step process for the biosynthesis of Ch-silver and Ch-gold nanocomposites using *Bacillus subtilis* bacterium. In another study, two marine fungi *Aspergillus* sp. Silv2 and *Alternaria* sp. Gol2 were used for the biological synthesis of Ch-silver and Ch-gold nanocomposites [72].

## 3. Characterization of Synthesized Ch-Coated Polymeric Silver and Gold Nanoparticles

**Figure 2:**
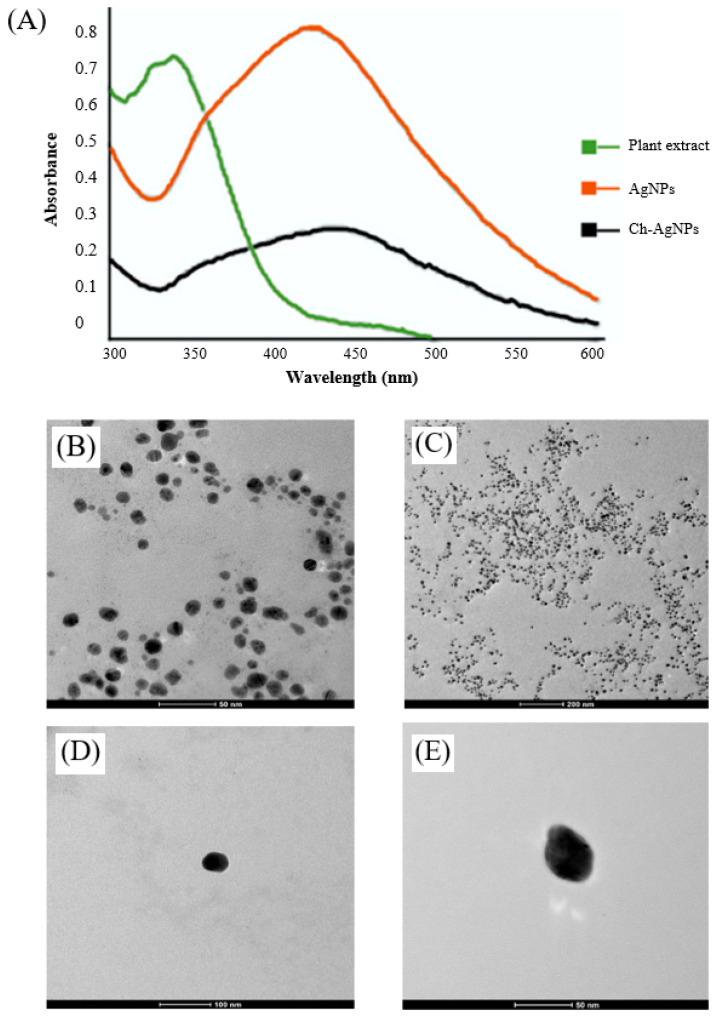
UV–Vis absorption spectra (**A**) and transmission electron microscopy analysis of synthesized AgNPs (**B**,**C**) and Ch-AgNPs (**D**,**E**). This figure has been reprinted with permission from Ref. [46], copyright 2021, MDPI.

The characterization of nanoparticles and their nanocomposites is necessary for evaluating their physical and chemical properties, such as size, shape, morphology, purity, particle crystallinity, surface chemistry, etc. Different instruments and techniques have been utilized to investigate the physical characteristics of the silver and gold nanoparticles, as well as Ch-coated polymeric silver and gold nanoparticles. The commonly used instruments are UV-visible spectrophotometry (UV-vis), transmission electron microscope (TEM), scanning electron microscope (SEM), X-ray diffraction (XRD), Fourier transform infrared spectroscopy (FTIR), and dynamic light scattering (DLS), etc. The synthesis of silver and gold nanoparticles and Ch-coated polymeric silver and gold nanoparticles are initially observed by the naked eye due to the color change. Generally, the brown or dark brown color of the reaction mixture indicates the synthesis of AgNPs and the Ch-coated polymeric silver nanocomposite, and the wine red, pink, violet, or purple color of the reaction mixture indicates the synthesis of AuNPs and the Ch-coated polymeric gold nanocomposite [1,27,46,47]. Then, the formation of AgNPs and AuNPs or Ch-coated polymeric silver or gold nanocomposite is confirmed by UV-visible spectrophotometry. Synthesized AgNPs and Ch-AgNPs showed a strong peak at around 400–500 nm in UV-visible spectrophotometry [1,2,46,58]. Similarly, synthesized AuNPs and Ch-AuNPs showed peaks at around 500–600 nm in UV-visible spectrophotometry [1,47]. The absorption spectra depended on the morphology, size, and shape of the synthesized nanoparticles [2,74]. According to Shinde et al. [46], the biosynthesized AgNPs and Ch-AgNPs using the leaf extract of *Prunus cerasus* showed an absorption peak at 429 and 445 nm, respectively (Figure 2A).

The morphology, shape, size, purity, and aggregation of Ch-coated polymeric silver or gold nanoparticles are observed by TEM and SEM analysis. Shinde et al. [46] utilized TEM to investigate the morphology, purity, and aggregation of biosynthesized Ch-AgNPs. The TEM analysis revealed that the spherical shape and silver nanoparticles were completely coated by Ch and showed a clear layer surrounding their core (Figure 2B–E). In another study by Paulkumar et al. [51], SEM was used to check the morphology of the biosynthesized Ch-AgNPs and found that the synthesized silver nanoparticles were strongly bound on the surface of the biopolymer Ch. X-ray diffraction is used to analyze the structural features of nanoparticles and nanocomposites, such as crystallinity, particle size, etc. [2]. DLS is used for the investigation of particle size distribution and polydispersity index. Shinde et al. [46] reported the average particles of biosynthesized AgNPs and Ch-AgNPs were 32.16 and 50 nm, respectively, with a polydispersity index of 0.2. They also investigated the zeta potential of biosynthesized AgNPs and Ch-AgNPs to check the stability of AgNPs and Ch-AgNPs in aqueous suspensions [46]. FT-IR analysis of nanoparticles and nanocomposites is performed to identify the available biomolecules responsible for the synthesizing and stabilizing of nanoparticles and nanocomposites [46]. The biosynthesis of Ch-AgNPs using fungal biomass and their characterization by UV–vis, SEM, energy dispersive X-ray analysis, DLS, and FTIR has been reported by Raza et al. [54]. Shinde et al. [46] have also reported the biosynthesis of Ch-coated AgNPs from the leaf extract of *Prunus cerasus* and the synthesized nanocomposites were analyzed by UV-Vis, TEM, FT-IR, DLS, and zeta potential analyzer.

**Figure 3:**
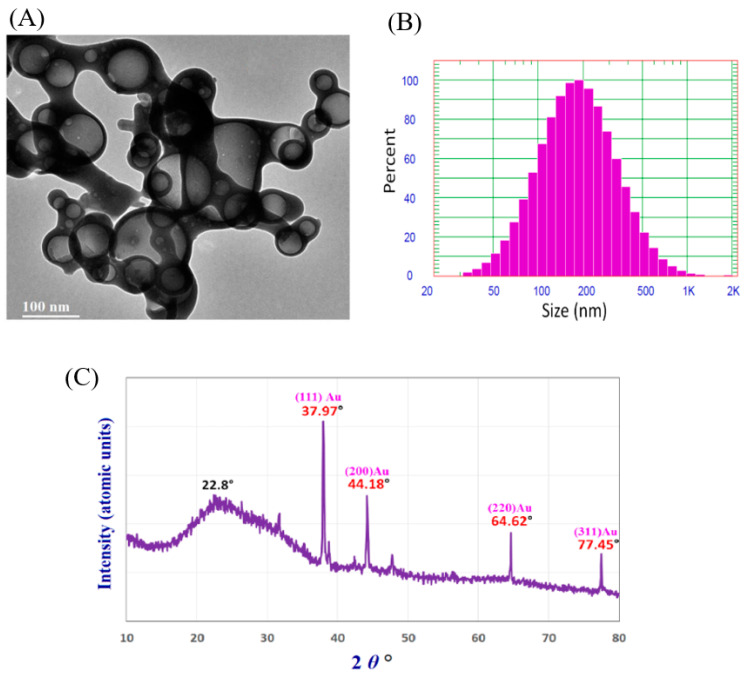
TEM images (**A**), particle size distribution (**B**), and XRD pattern (**C**) of Ch-AuNPs. This figure has been reprinted with permission from Ref. [36], copyright 2022, MDPI.

The biosynthesis of AuNPs and Ch-AuNPs using black pepper extract and their characterization by UV–vis, DLS, zeta potential, TEM, SAED, and EDX have been reported by Saha et al. [47]. The ecofriendly synthesis of Ch-AuNPs and their characterization by TEM, SEM, XRD, DLS, and FTIR have been conducted by Hashem et al. [36] (Figure 3). According to Hashem et al. [36], the TEM analysis showed a spherical shape with sizes ranging from 20 to 120 nm (Figure 3A). The DLS analysis showed that the average diameter of synthesized Ch-AuNPs was 218.2 nm (Figure 3B). The DLS analysis revealed the large size of synthesized Ch-AuNPs, compared to TEM analysis because of the presence of water molecules during DLS analysis around the synthesized Ch-AuNPs [36]. The XRD pattern of Ch-AuNPs revealed the crystalline nature of Ch-AuNPs (Figure 3C). The XRD pattern showed five clear diffraction peaks. Among these five diffraction peaks, the peak at 22.8° assured the presence of Ch in crystalline form. Other four peaks at 37.9°, 44.1°, 64.6°, and 77.4° confirmed the presence of AuNPs [36].

## 4. Potential Antibacterial Applications of Ch-Coated Polymeric Silver and Gold Nanoparticles

Bacterial resistance to various antibiotics is a serious problem worldwide. Numerous infections caused by multidrug-resistant bacteria are sometimes impossible to treat, leading to the death of many people worldwide [75]. According to the World Health Organization, at least 700,000 people are currently dying every year due to drug-resistant diseases and among these 700,000 people, 230,000 people die only from multidrug-resistant tuberculosis caused by a bacterium. If no action is taken, antimicrobial resistance could force up to 24 million people into extreme poverty by 2030 and drug-resistant diseases could cause 10 million deaths every year by 2050 [76]. Therefore, the development of novel, safe, and effective antibacterial agents to control multidrug-resistant bacteria and treat infectious diseases is urgently needed. Ch-coated polymeric silver and gold nanoparticles could be potential and effective antibacterial agents that solve these problems. The biopolymer Ch shows excellent antibacterial activities against numerous Gram-positive and Gram-negative pathogenic bacteria [29,30]. According to Avadi et al. [77], Ch showed strong antimicrobial activity against pathogenic *E. coli*. Costa et al. [78], investigated the antimicrobial activity of Ch against six oral pathogenic bacterial strains, such as *Prevotella buccae* (CCUG 15,401), *Tannarella forsythensis* (CCUG 51,269), *Aggregatibacter actinomycetemcomitans* (CCUG 13,227), *Streptococcus mutans* (CCUG 45,091), *Porphyromonas gingivalis* (9704 CIP 103,683T), and a clinical isolate of *Prevotella intermedia,* and found that the bioactive Ch effectively inhibits the growth of these pathogens with low MICs (minimum inhibitory concentrations) and shows quick and efficient bactericidal activity [78]. Jiang et al. [79] investigated the antimicrobial activity of two water-soluble chitosans against 31 representative foodborne pathogens and found that the used chitosans effectively controlled most of these foodborne pathogens. Many other studies also support the antimicrobial efficacy of Ch against different pathogenic Gram-positive and Gram-negative bacteria [29,80,81]. Similarly, many studies showed the efficacy of silver and gold nanoparticles in controlling various multidrug-resistant bacteria [1,82]. According to Huq [83], biosynthesized AgNPs using *Lysinibacillus xylanilyticus* MAHUQ-40 showed strong antimicrobial activity against drug-resistant human pathogenic bacteria *Vibrio parahaemolyticus* and *Salmonella* Typhimurium. Huq and Akter [12], also discovered the potential antimicrobial activity of bacterial-mediated synthesized AgNPs against multidrug-resistant pathogenic bacteria *K. pneumoniae* and *S.* Enteritidis. They used the disk diffusion method to investigate the zone of inhibition and microdilution assay to investigate the MICs and minimum bactericidal concentrations (MBCs) [12]. Hasnain et al. [45] reported on the panchagavya extract-mediated biosynthesis of AuNPs and investigated their antibacterial activity against *B. subtilis, E. coli*, and *K. pneumoniae*. They found that panchagavya extract-mediated biosynthesized AuNPs exhibited strong antibacterial activity against all these three pathogenic bacteria [84].

Silver and gold as metal exhibit toxicity even at a minimum concentration level [85]. The main properties of the bioactive polymer Ch are its nontoxicity, biodegradability, biocompatibility, low immunogenicity, and hemostatic properties [46,86,87,88]. By the conjugation of bioactive Ch with bioactive silver nanoparticles or gold nanoparticles, their efficacy and stability will increase significantly and the toxicity of silver and gold nanoparticles will decrease. According to Potara et al. [89], Ch stabilizes the AgNPs and prevents agglomeration. Ch also confers a positive charge to the surface of AgNPs, which enhances their binding to the negative charges present on the cell surface of bacteria [89]. According to Saha et al. [47], Ch increases the stability and efficacy of biosynthesized AuNPs. Shinde et al. [46] investigated the antibacterial activity of biosynthesized AgNPs and Ch-AgNPs and found that the biosynthesized Ch-AgNPs show high activity against pathogenic bacteria, compared to the biosynthesized AgNPs. They also found that Ch-AgNPs do not show any toxicity in normal cells. They used the leaf extract of *Prunus cerasus* for the biosynthesis of both AgNPs and Ch-AgNPs and evaluated their antimicrobial activity against multidrug-resistant pathogenic bacteria, such as *Enterococcus faecalis*, *E. coli*, *S. aureus*, and *K. pneumonia*. The results of this study demonstrated that the Ch-AgNPs could inhibit the growth of multidrug-resistant pathogenic bacterial strains more effectively than AgNPs alone [46]. Paulkumar et al. [51] reported the antibacterial activity of plant-extract-mediated Ch silver nanocomposites against several pathogenic bacterial strains, such as *Klebsiella planticola* (MTCC 2277), *B. subtilis* (MTCC 3053), *S. faecalis* (ATCC 8043), *E. coli* (ATCC 8739), and *P. aeruginosa* (ATCC 9027). The biosynthesized Ch-AgNPs show strong antibacterial activity against all the tested pathogens, and they demonstrated that the silver-based Ch nanocomposite shows potent antibacterial activity due to the presence of small-sized silver nanoparticles on the surface of Ch [51]. Saruchi et al. [59] used the plant extract of *Saccharum officinarum* for the green synthesis of Ch-AgNPs and the synthesized nanocomposite was used to control the pathogenic *B. cereus*, *Staphylococcus*, and *E. coli*. They found that the synthesized bionanocomposites are potentially very effective against all tested pathogenic strains of bacteria and concluded that the biosynthesized Ch–silver nanocomposite could be a drug potentially used to control various pathogenic bacteria [59]. Fuster et al. [27], investigated the antibacterial activity of Ch-AuNPs against Gram-negative *E. coli* ATCC 25,922 and a clinical isolate of *E. coli* 11,046 (CI-EC) and two Gram-positive bacterial strains, methicillin-sensitive *S. aureus* ATCC 29213 and methicillin-resistant *S. aureus* ATCC 43,300. The Ch-AuNPs displayed significant antibacterial activity against all tested pathogenic strains, suggesting that Ch-AuNPs could be promising nanostructures for reducing bacterial infections [27].

**Figure 4:**
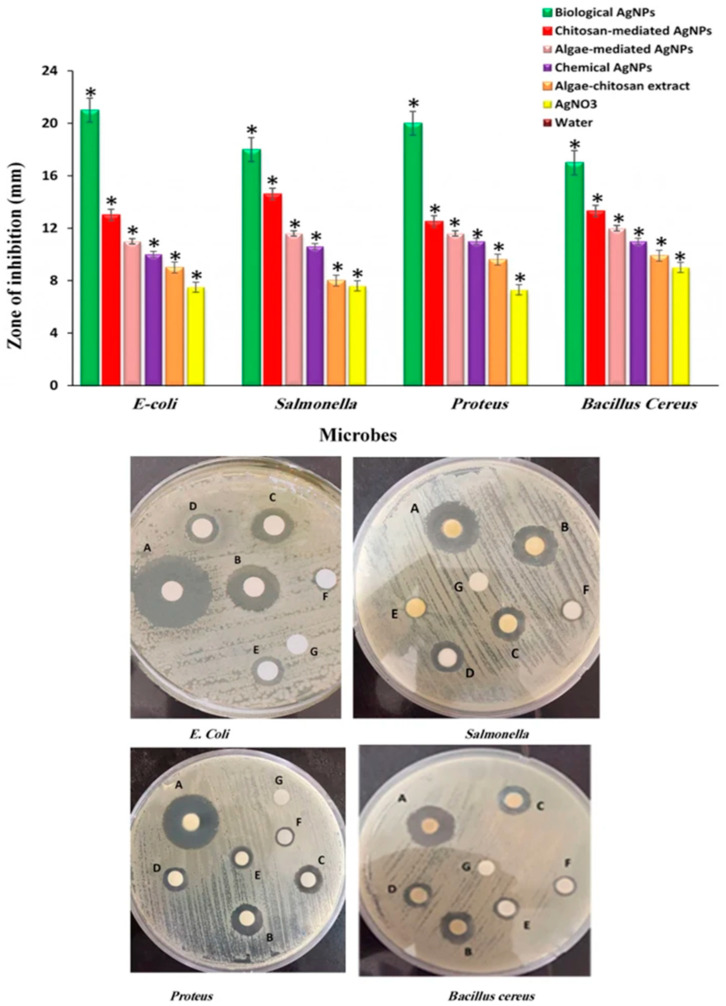
Comparative antibacterial effect of Ch-coated AgNPs against four selected clinical pathogens. A, biological AgNPs; B, Ch-mediated AgNPs; C, algae-mediated AgNPs; D, chemical AgNPs; E, raw extract; F, AgNO_3_; and G, H_2_O. This figure has been reprinted with permission from Ref. [89], copyright 2020, Nature Portfolio.

Rezazadeh et al. [90] synthesized different AgNPs, including biogenic Ch-AgNPs, and investigated their antibacterial efficiency against four pathogenic bacterial strains (*E. coli*, *Proteus*, *Salmonella*, and *B. cereus*) using the disk diffusion method. The antibacterial activity of different AgNPs, such as algae-extract-mediated Ch-AgNPs (biological AgNPs), algae-extract-mediated AgNPs (algae-mediated AgNPs), only-Ch-mediated AgNPs, chemically synthesized AgNPs (chemical AgNPs), and AgNO_3_ solution are shown in Figure 4 [90]. The results showed that the algae extract-mediated Ch-AgNPs (biological AgNPs) exhibit superior effectiveness against all four selected bacterial strains, compared to all other AgNPs, algae-Ch extract, and AgNO_3_ precursor (Figure 4) [90]. The algae-extract-mediated Ch-AgNPs showed the largest zone of inhibition against four tested pathogenic bacterial strains, which were 21, 20, 18, and 17 mm, against *E. coli*, *Proteus*, *Salmonella*, and *B. cereus*, respectively, (Figure 4). The marine algae extract contains various biomolecules, which encompass the surface of biological AgNPs. When these bioactive AgNPs are coated by biopolymer Ch, the biological applicability and biocompatibility of Ch-AgNPs would presumably enhance, and hence increase, the antibacterial properties [90].

## 5. Antibacterial Mechanisms of Ch-Coated Polymeric Silver and Gold Nanoparticles

The antibacterial activity of Ch-coated polymeric silver or gold nanoparticles largely depends on the type of Ch, molecular weight of Ch, type or size of silver or gold nanoparticles, molecular ratio of Ch and silver or gold nanoparticles, and the synthesis conditions, such as pH, temperature, etc. [2,27]. The positively charged Ch and silver or gold nanoparticles provide antibacterial properties because of their interaction with the negatively charged cell membranes of both Gram-negative and Gram-positive bacteria [2,27]. The complete antibacterial mechanism of Ch-coated polymeric silver or gold nanoparticles is still not fully known. There are several proposed mechanisms for the antibacterial activity of Ch against Gram-negative and Gram-positive bacteria. The most acceptable mechanism is the interaction between positively charged Ch molecules (NH^3+^ groups) and negatively charged bacterial cell membranes, producing changes in the membrane permeability, which cause osmotic imbalances, inhibit bacterial growth and hydrolysis of the cell wall peptidoglycans of bacteria, and, finally, lead to the leakage of intracellular electrolytes, including potassium ions, as well as amino acids and low-molecular-weight proteins [27,91]. According to Sebti et al. [92], after penetrating the Ch into the nuclei of the bacteria through the cell wall, the Ch makes bonds with microbial DNA, which inhibits the synthesis of mRNA and protein and halts the normal activity of the cell. Another mechanism is the chelation of essential microbial nutrients with Ch [93]. According to Wang et al. [94], Ch has excellent metal-binding capacities, which influence the binding of different essential metallic nutrients with Ch in the bacterial cell that inhibit the growth of bacteria.

**Figure 5:**
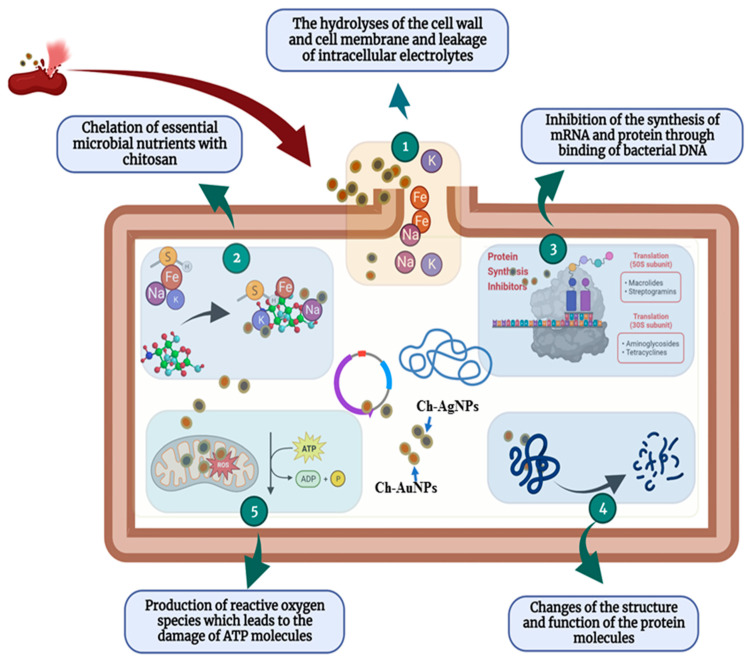
Probable antibacterial mechanisms of chitosan-coated silver and gold nanoparticles.

The combination of Ch and silver or gold nanoparticles seems promising because the positively charged bioactive polymer Ch potentiates interactions with bacteria, enhancing the positively charged silver or gold nanoparticles to disrupt the bacterial cell membrane more successfully. In this way, biopolymer Ch increases the biocompatibility and antibacterial activity of silver or gold nanoparticles [94,95]. The positively charged silver ions interact with the cell membrane of bacteria, disturbing the membrane permeability and respiration, as well as interacting with the negatively charged DNA and protein molecules, which could collapse the structure and function of DNA and protein [2,51]. The release of free radicals from silver might also be involved in membrane damage [51]. According to Fuster et al. [27], the antibacterial mechanism of Ch-AuNPs involves the electrostatic interactions between the Ch-AuNPs and the bacterial cell membranes. These interactions lead to structural modification and loss of the properties of the bacterial membrane. Although the exact antibacterial mechanism of Ch-coated silver or gold nanoparticles has not been thoroughly explained, the probable antibacterial actions of Ch-coated silver or gold nanoparticles have been proposed in Figure 5. The proposed antibacterial mechanisms of Ch-coated silver and gold nanoparticles are the hydrolyses of the cell wall and cell membrane, the leakage of intracellular electrolytes and low-molecular-weight proteins, chelation of essential microbial nutrients with Ch, inhibition of the synthesis of mRNA and protein through the binding of bacterial DNA, alteration of the structure and function of the protein molecules, and the production of reactive oxygen species, which leads to the damage of ATP molecules (Figure 5). Through the above possible mechanisms, Ch-coated silver and gold nanoparticles inhibit the growth of pathogenic bacteria and finally kill them.

## 6. Conclusions and Future Perspectives

The emergence of MDR pathogenic bacteria is a serious threat to public health worldwide. Therefore, the development of safe and effective antibacterial agents is urgently required. Ch-coated polymeric silver and gold nanoparticles represent an emerging group of bioactive hybrid materials in medical science because of their biodegradability, biocompatibility, high activity, and stability with low toxicity. Ch is a biopolymer and non-toxic polymer that shows excellent antibacterial activities against numerous pathogenic microorganisms. Similarly, biosynthesized AgNPs and AuNPs also exhibit strong antimicrobial activity against numerous pathogenic microorganisms, including multidrug-resistant bacteria. By the conjugation of Ch with AgNPs or AuNPs, the stability of Ch-coated silver or gold nanoparticles will be increased and toxicity will be decreased, as well as the efficacy being increased significantly. Some recent studies showed the high stability and improved efficacy of Ch-coated silver and gold nanoparticles against pathogenic bacteria. In this review, the facile, non-toxic, and eco-friendly method for the biosynthesis of Ch-coated polymeric silver or gold nanoparticles and their characterization have been comprehensively reviewed. The antibacterial applications and mechanisms of the biosynthesized Ch-coated polymeric silver or gold nanoparticles against pathogenic bacteria have also been highlighted. Although the biosynthesized Ch-coated polymeric silver or gold nanoparticles have shown great potential in controlling MDR pathogenic bacteria, several points might be considered for the future biosynthesis of Ch-coated polymeric silver or gold nanoparticles to explore their potent antibacterial activity. First, the type and molecular weight of Ch, the concentration of Ch, the concentration of silver or gold salts, and the concentration of plant or microbial extracts. These factors not only influence the synthesis process but also influence the antibacterial activity. Second, the biosynthesis of Ch-coated silver or gold nanoparticles should be conducted using potential and available plants or microbes, such as medicinal plants or other pharmacologically active plants and beneficial microbes or probiotics. Third, optimum synthesis conditions, i.e., temperature, pH, time, etc., should be maintained. Fourth, the investigation of the cytotoxic effect of biosynthesized Ch-coated polymeric silver or gold nanoparticles on human cells is essential, though some studies suggest that Ch-coated polymeric silver or gold nanoparticles are non-toxic and safe to use. Finally, it can be said that Ch-coated polymeric silver and gold nanoparticles could be a promising tool in nanomedicine for controlling multidrug-resistant pathogenic bacteria.

## Figures and Tables

**Table 1:** Biosynthesis of chitosan-coated polymeric silver and gold nanoparticles and their potential antibacterial applications.

Nanoparticles	Synthesis Method	Characterization of Synthesized Nanoparticles	Applications	References
Ch-silver	Green synthesis of Ch silver bionanocomposite using the plant extract of *Saccharum officinarum*.	Characterized by UV-vis spectrophotometer, TEM, and FTIR.	Antibacterial applications against *Bacillus cereus*, *Staphylococcus*, and *Escherichia coli*.	[59]
Ch-silver	Synthesis of Ch silver bioconjugates using leaf extract of *Prunus cerasus*.	The bioconjugates were characterized using TEM, DLS, FT-IR, UV–Vis spectroscopy, and a zeta potential analyzer.	Antimicrobial applications *E. coli*, *Enterococcus faecalis*, *Klebsiella pneumoniae*, and *S. aureus*.	[46]
Ch-silver	Biosynthesis of Ch silver nanocomposite using Aloe vera extract and *Cuscuta reflexa* extract.	Characterized by UV–vis spectrum, FT-IR, and SEM	Antibacterial activities against *Staphylococcus aureus* ATCC 33592, *K. pneumoniae* ATCC 13884, *Bacillus subtilis* ATCC 55614, and *E. coli* ATCC 11229.	[58]
Ch-silver	Green synthesis of Ch silver nanoparticles using vitamin C as a reducing agent.	SEM, Zeta potential, and XRD.	In vitro antimicrobial activities against *E. coli and S*. Typhimurium, and in vivo antibacterial activity against *E. coli* in minced beef meat samples.	[60]
Ch-silver	Green synthesis of Ch-AgNPs using Ch as a stabilizer and sodium hydroxide as a reducing agent.	UV–vis spectroscopy, FT-IR spectroscopy, XRD, SEM, EDX, and zeta sizer nano.	Antibacterial activity against *S. aureus*, *E. coli*, and antifungal activity against *Candida albicans*.	[61]
Ch-silver	Green synthesis by a simple and environmentally friendly in situ chemical reduction process.	UV–Vis, TEM, SEM, XRD, and FTIR.	Antibacterial activity against *S. aureus*, and *E. coli*.	[26]
Ch-silver	Green and rapid synthesis of Ch-AgNPs using economically abundant biopolymer crustacean waste.	UV–visible spectral, FTIR, XRD, AFM, TEM, and DLS.	Antibacterial activity against *Bacillus* sp., *Staphylococcus* sp., *Pseudomonas* sp., *E. coli*, *Proteus* sp., *Serratia* sp. and *Klebsiella* sp. Antifungal activity against *Aspergillus niger*, *A. fumigatus*, *A. flavus*, and *C. albicans*.	[62]
Ch-silver	Biosynthesis of AgNPs and Ch-AgNPs using the stem extract of *Saccharum officinarum*.	Characterized by UV–vis, TEM, SEM, and FTIR.	Antibacterial activity against *B. subtilis* (MTCC 3053), *K. planticola* (MTCC 2277), *Streptococcus faecalis* (ATCC 8043), *P. aeruginosa* (ATCC 9027), and *E. coli* (ATCC 8739).	[51]
Ch-silver	Biosynthesis of Ch-AgNPs using fungal biomass (*Aspergillus fumigatus* KIBGE-IB33).	UV–vis, SEM, DLS, and FTIR.	Antimicrobial activity against *Enterococcus faecalis* ATCC 29212 *S*. Typhimurium ATCC 3632, *Listeria monocytogenes* ATCC 7644, and *P. aeruginosa* ATCC 27853.	[54]
Ch-silver	Green synthesis of Ch-AgNPs using Ch as a reducing agent, as well as the stabilizing agent.	UV–Vis, FTIR spectroscopy, TEM, XRD, and DLS.	Antibacterial activity against Gram-positive *S. aureus* (KMIEV B161), and Gram-negative *E. coli*.	[63]
Ch-silver	The green route was used for the synthesis of Ch-based silver nanoparticles using Ch as a reducing and stabilizing agent.	UV–vis, FTIR, SEM, XRD, and TEM.	Antibacterial activity against *P. aeruginosa*, *E. coli*, and methicillin-resistant *S. aureus*.	[64]
Ch-silver	Ch-AgNPs were synthesized using AgNO_3_, cysteine, and Ch.	UV–vis, DLS and Zeta potential, TEM, and XRD.	Antifungal activity against *Sporothrix brasiliensis*, and *Sporothrix schenckii*.	[65]
Ch-silver	Biosynthesis of Ch-AgNPs using leaf extract of *Cissus arnottiana*.	UV–Vis, SEM, TEM, AFM (atomic force microscope), XRD, and SAED.	Antibacterial and antifungal activity against *S. aureus*, *Streptococcus mutans*, *E. faecalis*, and *C. albicans*.	[53]
Ch-silver	Ch ascorbic acid-based green synthesis of polymeric silver nanoparticles.	UV–Vis, TEM, X-ray photoelectron spectroscopy (XPS).	Antibacterial and antifungal activity against *S. aureus*, *P. aeruginosa*, *E. coli*, and *C. albicans*.	[66]
Ch-silver	Green synthesis of Ch-AgNPs using Ch as a reducing agent, as well as the stabilizing agent.	UV–vis, FTIR, XRD, and high-resolution transmission electron microscopy (HRTEM).	Catalytic activity and antibacterial activity against *E. coli*, and *M. luteus*.	[67]
Ch-silver	Synthesis of biogenic Ch-AgNPs using Ch as a reducing agent, as well as the stabilizing agent.	UV–vis, FTIR, EDX, SEM, TEM, and XRD.	Anticancer activity in human hepatocellular carcinoma HepG2 cells.	[68]
Ch-silver	Biogenic synthesis of Ch functionalized silver nanoparticles using leaf extract of *Carica papaya*.	UV–vis, FTIR, DLS, HRTEM, and zeta potential estimation.	Antibacterial and antibiofilm activities against *E. coli*, and *S. aureus*.	[69]
Ch-silver	Biosynthesis of AgNPs and Ch-AgNPs using seed extract of *Piper nigrum*.	UV–vis, XRD, SEM, TEM, and FTIR.	Antibacterial activity against *E. coli*, and *Bacillus subtilis*.	[70]
Ch-silver and Ch-gold	Biosynthesis of Ch-silver and Ch-gold nanocomposites using *Bacillus Subtilis*.	UV–vis, XRD, SEM, and TEM.	Antibacterial activity against *S. aureus*, and *P. aeruginosa*. Antifungal activity against *A. niger*, and *C. albicans*.	[71]
Ch-silver and Ch-gold	Biosynthesis of Ch-silver and Ch-gold nanoparticles using two endophytic fungi, *Aspergillus* sp., and *Alternaria* sp.	UV–vis, XRD, FTIR, and TEM.	Antibacterial activity against *E. coli*, and *S. aureus*. Antibiofilm activity against *P. aeruginosa*, *B. subtilis*, *E. coli*, and *S. aureus*.	[72]
Ch-gold	The AuNPs and Ch-AuNPs have been biosynthesized using the extract of black pepper (*Piper nigrum*)	UV–vis, DLS, zeta potential, TEM, SAED, and EDX.	Antifilarial activity against *Setaria cervi* causes filarial parasite disease.	[47]
Ch-gold	Ch-AuNPs were synthesized using gold (III) chloride trihydrate and Ch.	UV–Vis, FE-TEM, FE-SEM, Zeta potential, and EDX.	Antifungal activity against *C. albicans*.	[73]
Ch-gold	Green synthesis of Ch-AuNPs using Ch as a reducing and stabilizing agent.	UV–vis, DLS, and TEM.	Antibacterial activity against *S. aureus* ATCC 29213, *S. aureus* ATCC 43300, and *E. coli* 11046.	[29]
Ch-gold	Green synthesis of Ch-AuNPs using Ch as a reducing and stabilizing agent.	TEM, SEM, FTIR, and XRD.	Antibacterial activity against *P. aeruginosa*, and *S. aureus*. Antifungal activity against *C. albicans*.	[36]

## Data Availability

Not applicable.
